# Juvenile hormone analog enhances Zika virus infection in *Aedes aegypti*

**DOI:** 10.1038/s41598-021-00432-1

**Published:** 2021-10-26

**Authors:** Abdullah A. Alomar, Bradley H. Eastmond, Barry W. Alto

**Affiliations:** Department of Entomology and Nematology, Florida Medical Entomology Laboratory, University of Florida, Vero Beach, FL 32962 USA

**Keywords:** Ecology, Infectious diseases, Virus-host interactions

## Abstract

In recent years, there has been a rise in the emergence of arboviruses of public health importance, including Zika, chikungunya, dengue, and yellow fever viruses. Insecticide-based mosquito control has been the primary method for mitigating transmission of arboviruses. The consequences for the application of insecticides include both lethal and sublethal effects, and associated development of insecticide resistance. However, little is known about the influence on arboviral transmission. Mosquitoes with phenotypes that exhibit insecticide resistance or experience sublethal effects may be associated with altered susceptibility to arbovirus infection and transmission. Juvenile hormone analogs (JHAs) are insecticides that prevent pupa to adult molting of mosquitoes by mimicking the action of their natural juvenile hormone. Here, we examined whether the JHA pyriproxyfen interacts with ambient temperature (20 °C and 30 °C) during juvenile stages to influence life-history traits, population growth (λ'), and Zika virus (ZIKV) infection in *Aedes aegypti*. Development time of females was lengthened at 20 °C and in the presence of JHA. Prevention of pupa to adult molting by JHA was differentially higher at elevated temperature than low temperature. Size of females was larger at 20 °C and smaller at 30 °C. Infection, disseminated infection, and transmission of ZIKV in females were enhanced by JHA at both 20 °C and 30 °C relative to the controls. These results demonstrate that mosquito life-history and vector competence parameters are strongly influenced by interactive effects of JHA and temperature. The JHA-induced enhancement of ZIKV infection in females should be a consideration when implementing JHA in vector control strategies.

## Introduction

The widespread deployment of insecticides for control of insect vectors of human pathogens has been in place for the better part of a century. The development of insecticide resistance is a common consequence of control practices that deploy insecticides into the environment^1, 2^. Reduction of insecticide use and rotation practices that make use of different insecticides without cross-resistance properties are methods employed to manage insecticide resistance^3, 4^. Those mosquitoes that avoid the lethal effects of insecticides, perhaps attributable to being resistant or having been exposed to a sublethal dose are of particular concern from a public health perspective. Specifically, these mosquitoes likely comprise a major portion of the adult population following insecticide treatment in areas most at risk for transmission and human infection. Studies to date on interactions between mosquitoes and arboviruses have not adequately evaluated altered phenotypes of these mosquitoes. Specifically, altered phenotypes of mosquitoes may inhibit, enhance, or have inconsequential effects on vector competence for pathogens, as well as other traits related to the ability of mosquitoes to transmit pathogens. For example, insecticide resistance genes may be associated with pleiotropic effects on phenotypic traits, including vector competence, which describes susceptibility to infection and transmission of pathogens^5, 6^. Sublethal exposure to insecticides may alter mosquito immune responses governing vector competence^7–10^. The ecological consequences of such effects remain largely unexplored and critically important to consider as parameters of models assessing risk of pathogen transmission.

The efficacy of insecticides is likely influenced by environmental temperature, and perhaps more so for resistant mosquitoes and mosquitoes that are exposed to sublethal doses. Juvenile hormone analogs (JHAs) are alternative insecticides for mosquito control and they are favorable candidates for control, due to lower rates of resistance, and due to their mode of actions. Juvenile hormone analog pyriproxyfen is a chemical growth regulator that mimics the action of natural juvenile hormone (JH) in insects and disrupts the insect endocrine system and inhibits pupa-adult molting^11^. The presence of JH in insects is necessary for the maintenance of the juvenile status, whereas the absence of JH is a prerequisite for pupa-adult molting to occur^12, 13^. The application of JHA at juveniles can therefore alter the hormonal balance and prevent pupa-adult molting, resulting in death among pupae^14^.

Several empirical studies showed a powerful effect of JHAs in preventing mosquito pupa-adult molting following juvenile exposure^15–19^. The use of autodissemination stations through a pull–push pest management approach (i.e., attraction and transfer followed by dispersal to targets) lures mosquitoes to a station where they become tainted with JHA, and then disseminate JHA to other larval habitats. Deployment of JHA to target mosquitoes is an effective strategy of control that can induce juvenile mortality as a lethal effect^20–22^. In addition to lethal juvenile mortality, sublethal effects of JHA have been observed to cause morphological, physiological, and behavioral changes among juveniles and adults^16^. For instance, exposure to JHA changed swimming behavior and caused damages in larval midgut cells of *Ae. aegypti*^23^. Reproduction disruption and longevity reduction of adult *Ae. aegypti* were observed following JHA exposure^19, 24, 25^. The results of these studies, and others, suggest that JHA can modify mosquito traits, including immunity and susceptibility to pathogen infection among surviving adults as detected with other insecticides^7, 18, 26^.

Juvenile hormone analogs can be applied to reduce mosquito population under a variety of environmental conditions in which there is a risk of transmission of arboviruses. Temperature is a significant abiotic environmental factor that plays a key role in determining mosquito development and responses to pathogen infection^27^. An interaction between temperature and JHA is possible since the latter acts on physiological processes (i.e., mimics JH) essential for mosquito growth and development, life-history traits highly dependent on temperature^28, 29^. Several studies found modifications of JHA effects on mosquitoes under different temperatures. For instance, JHA-induced mortality in juvenile *Culex pipiens* was enhanced under elevated temperature compared to cooler temperature^30^. The efficacy of larval exposure to JHA in suppressing juvenile and adult survival in *Ae. aegypti* was altered under different temperature regimes^31^. These observations suggest that temperature may interact with JHA to influence mosquito life-history traits and other biological processes of adults relevant to infection with pathogens. We employ an insect vector study system using *Ae. aegypti*, the primary vector of several emerging arboviruses with public health importance. This species is an invasive mosquito, native to Africa, and is widely distributed in tropical and subtropical regions globally^32^. *Aedes aegypti* is adapted to human-dominated areas which can increases the chance of acquiring and transmitting arboviruses among humans^32^. We also make use of infection studies using Zika virus (ZIKV), which until recently was a relatively obscure virus in Africa until its outbreak occurred in French Polynesia and South America in 2013 and 2015, respectively, leading to declaration of a public health emergency of international concern by the World Health Organization in 2016^33, 34^. The aim of our study was to assess the interactive effects of JHA and temperature on juveniles and adults of *Ae. aegypti* life-history traits, population growth, and responses to ZIKV infection.

## Methods

### Origin and rearing of mosquitoes

Wild-type *Ae. aegypti* mosquitoes were collected from containers in the City of Vero Beach, Florida and propagated in laboratory until third generation. Larvae were reared at 28 °C and 70% relative humidity with a 14-h light-10-h dark photoperiod and fed larval food comprised of an equal mixture of lactalbumin and *Saccharomyces cerevisiae* yeast. Adults were fed on 10% sucrose solution delivered via cotton balls. To generate eggs, females were blood-fed on chickens following Animal Use Protocol (202007682) approved by University of Florida's Institute of Animal Care and Use Committee. All methods were carried out in accordance with ARRIVE (Animal Research: Reporting of in vivo Experiments) and other relevant guidelines and regulations. Vertebrate animals were only used in producing mosquito eggs and therefore did not influence study design, experimental procedures, and downstream sampling of mosquitoes. Blood‐engorged females were allowed to lay their eggs inside cups of water with the interior walls covered with germination paper. We exposed newly hatched larvae to several concentrations of JHA pyriproxyfen (Nyguard) (0.01, 0.0125, 0.015, 0.02, 0.03, 0.04, 0.05, 0.1, 0.2 ppb) to estimate the concentration that prevents 50% of pupa-adult molting before starting the experiment.

### Juvenile hormone analog and temperature

**Figure 1:**
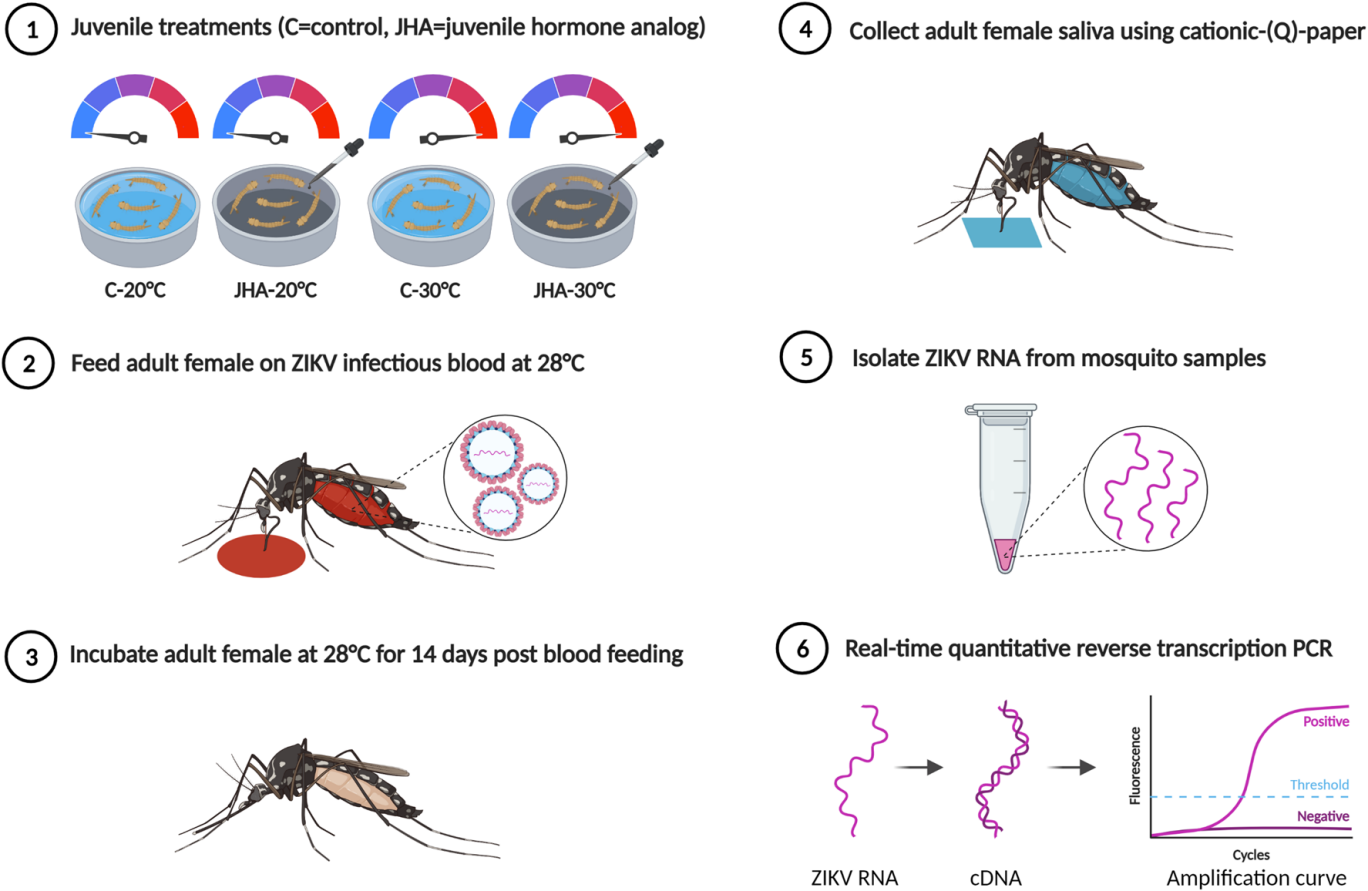
A diagram illustrating the experimental workflow.

Generation F_3_ *Ae. aegypti* eggs were hatched in tap water and 300 newly hatched larvae (< 24-h old) were added to containers with 1.5 L of tap water and fed 0.2 g of larval food. Containers with larvae were incubated at two constant temperatures (20 °C and 30 °C) using separate environmental chambers. For each temperature, a 0.03 ppb of JHA that causes approximately 50% pupa-adult molting prevention was applied to treatment groups, whereas control groups received no JHA application (Fig. 1). Five replicates were performed for each treatment. Supplemental larval food was added every 4 days. Pupae from each replicate were collected daily and placed in cages for adult emergence. Newly emerged adults from all treatments were maintained at 28 °C and fed on 10% sucrose solution. Juvenile development time (number of days from hatching to adulthood) and pupa-adult molting (survival to adulthood expressed as a percent of the original cohort) were recorded for each replicate.

### Cell culture, Zika virus propagation, and mosquito infection

Epithelial (Vero) cells of African green monkey *Cercopithecus aethiops* (American Type Culture Collection, Manassas, VA) were grown in growth media (M199) (HyClone, Medium 199, GE Healthcare, Logan, UT) supplemented with 10% heat-inactivated fetal bovine serum (Thermo Fisher Scientific, Waltham, MA), antibiotics (penicillin–streptomycin), and Mycostatin to confluence in T-175 cm^2^ cell culture flasks at 37 °C and 5% CO_2_ atmosphere. Asian-lineage ZIKV from Puerto Rico (strain PRVABC59, GenBank: KU501215.1) was obtained from a human clinical sample and provided by the U.S. Centers for Disease Control and Prevention (Division of Vector-Borne Diseases, Arboviral Diseases Branch). Cell monolayers were infected with ZIKV at a multiplicity of infection of 0.01 viruses per host cell and incubated for 1-h at 37 °C, rocking every 15-min to promote viral attachment, after which 24 ml of M199 were added, followed by a further incubation at 37 °C. Six days post-infection, virus-infected cell monolayers were harvested and combined with bovine blood (Hemostat Laboratories, Dixon, CA) and adenosine-5'-triphosphate disodium salt trihydrate (ATP, Thermo Fisher Scientific, Waltham, MA) to prepare the ZIKV infectious blood to challenge adult mosquitoes.

Adult females (7 to 10 days after emergence) were placed in cylindrical cages (0.5 L) 1 day before challenging them with ZIKV using artificial feeders (Discovery Workshops, Lancashire, UK) heated to 37 °C for 1-h after 24-h of starvation. Following oral feeding, mosquitoes were anesthetized with carbon dioxide and fully-fed individuals were transferred to clean cages with access to 10% sucrose solution, whereas partially-fed and unfed individuals were discarded. Infectious blood contained viral loads of 5.9 ± 0.1 log_10_ plaque-forming unit equivalents per ml (PFUE/ml). Adult females were held at 28 °C for 14 days post blood feeding which exceeds the theoretical median time from ingestion of ZIKV to transmission (EIP_50_, extrinsic incubation period)^35^. The temperature treatments deliberately isolate those effects on the juvenile stages, independent of temperature effects of virus infection in adults.

### Saliva and cationic-(Q)-paper

Mosquito saliva was collected from ZIKV infected females after 14 days post blood feeding using a cationic-(Q)-paper (CQP) approach^36^. Females were anesthetized with carbon dioxide and placed individually in *Drosophila* cultivation tubes (Thermo Fisher Scientific, Waltham, MA) containing CQPs that were treated with honey and blue dye. The blue dye was used as a visual marker to determine if a female fed on honey and deposited saliva during feeding^37, 38^. Females were allowed to orally feed on the CQPs for 3-h after 24-h of starvation. Following CQP assays, females were dissected to remove their legs and wings from the bodies. Whole bodies, legs, CQPs were separately placed into 2 ml microcentrifuge tubes (Thermo Fisher Scientific, Waltham, MA) containing incomplete M199 (1 ml) and kept at -80 °C until use. Collecting mosquito saliva through feeding on CQP was used as a proxy for transmission of ZIKV by a bite. The saliva was tested for mosquitoes that successfully fed on CQPs as indicated by visualization of blue coloring in their crop.

### Zika virus RNA isolation, cDNA synthesis, and real-time quantitative reverse transcription PCR

Mosquito body, legs, and CQP containing saliva were thawed and homogenized using a TissueLyser II automation system (Qiagen, Hilden, Germany) for 3-min at 19.5 Hz and centrifuged for 5-min at 13,200 rpm. Isolation of viral RNA from mosquito tissues and CQPs was performed using QIAamp Viral RNA Mini Kit (Qiagen, Germantown, MD, USA) and eluted in buffer (60 μl) according to the instructions provided by the manufacturer and stored at -80 °C until use^18^. Primers and probe (Integrated DNA Technologies, Coralville, IA) used in this study were as follows:

Forward primer (5′-CTTCTTATCCACAGCCGTCTC-3′).

Reverse primer (5′-CCAGGCTTCAACGTCGTTAT-3′).

Probe (5′-/56 FAM/AGAAGGAGACGAGATGCGGTACAGG/3BHQ_1/-3′).

The temperature profile of the qRT-PCR was 94 °C for 2-min, 94 °C for 12-s, 50 °C for 30-min, and 58 °C for 1-min. Susceptibility to ZIKV infection, disseminated infection, and transmission rates were determined by quantifying virus in bodies, legs, and saliva of mosquitoes, respectively, following ZIKV RNA isolation. The viral loads of ZIKV in mosquito tissues (body, legs, saliva) were quantified using a standard curve that compares cDNA synthesis to a range of ZIKV serial dilutions in parallel with plaque assays of the same dilutions of the virus, expressed as PFUE/ml^39^. We defined the infection and disseminated infection and transmission rates as the percentages of mosquitoes tested (fed) that had virus in their body, legs, or saliva, respectively.

### Size of infected mosquito

A single wing from each infected adult female was mounted on glass microscope slides (Cardinal Health, Dublin, OH) and length was measured in millimeters using computer imaging software (IMT i-Solution lit, Princeton, NJ) and a phase contrast microscope. Measuring wing length was used as a proxy for size of adult females^40, 41^.

### Finite rate of increase estimation (population growth)

An estimated finite rate of increase (*λʹ*) was calculated for each replicate container as:

$$\lambda^{{\prime }} = \exp \left( {r^{{\prime }} } \right) = \exp \frac{{In\left[ {\left( \frac{1}{No} \right)\sum xAxf\left( {Wx} \right)} \right]}}{{D + \left[ {\frac{{\sum x \times Axf\left( {Wx} \right)}}{{\sum xAxf\left( {Wx} \right)}}} \right]}}$$

where *No* is the initial number of females in the cohort (assumed to be 50%); *Ax* is the number of females emerged to adults on day *x*; *D* is the time from female emergence to adulthood to reproduction, taken as 12 days for *Ae. aegypti* based on the literature^42^; *f* (*Wx*) is a function based on the relationship between size and egg production in female mosquitoes. For *Ae. aegypti*, *f* (*Wx*) = 1/2(40.694*Wx*–48.739)^43^. Thus, we obtain fecundity estimates (number of eggs) based on mosquito size (wing length). The current experiment performed direct measurements of emergence to adulthood (pupa-adult molting), development time, and size of mosquitoes.

### Statistical analyses

Separate factorial multivariate analysis of variance tests (MANOVAs) were used to determine the treatment effects on life-history traits (development time, pupa-adult molting, female size) and vector competence measurements (infection, disseminated infection, and transmission). Standardized canonical coefficients (SCCs) were used as ranking scores for contributors to their respective function. Multivariate pairwise comparisons with sequential Bonferroni adjustment for experiment-wise alpha (0.05) were carried out to compare treatments following the detection of significant effects. The effects of treatment on *λ’* was analyzed using factorial ANOVA.

### Ethical statement

Experimental infection of mosquitoes with ZIKV was conducted in an arbovirology research facility (BSL2/3) at the Florida Medical Entomology Laboratory in accordance with the approved protocol by the University of Florida’s Institutional Biosafety Committee. The study conforms to appropriate regulations and guidelines set by the Institutional Animal Care and Use Committee (protocol 202007682).

## Results

### Life-history traits and population growth (λʹ)

**Figure 2:**
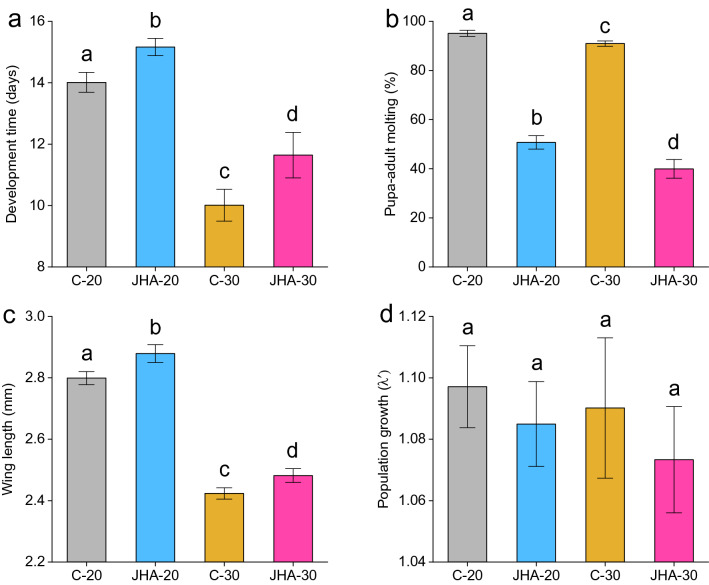
Treatment effects on juvenile development time (**a**), pupa-adult molting (**b**), female wing length (**c**), and population growth (λʹ) (**d**). Bars represent means ± standard error of the means. Means with different letters indicate statistically significant differences (*P* < 0.05) between each other.

Factorial MANOVA showed significant effects of JHA, temperature, and their interaction on juvenile development time, pupa-adult molting, and female wing length (size) (Table 1). Juvenile hormone analog exposure reduced pupa-adult molting, lengthened juvenile development time, and was associated with larger adults at both temperatures (Fig. 2a, b, c). Elevated temperature accelerated juvenile development time and resulted in smaller-sized adults (Fig. 2a, c). For the interaction, SCCs showed that pupa-adult molting contributed the most to the multivariate effect followed by development time and wing length (Table 1). Under low temperature, juvenile development time was significantly increased in comparison to elevated temperature (Fig. 2a). Prevention rate of pupa-adult molting by JHA was significantly enhanced at 30 °C comparing to 20 °C (Fig. 2b). At 20 °C, females had larger wing length comparing to those individuals derived from 30 °C (Fig. 2c). Factorial ANOVA showed that *λ’* was not significantly affected by JHA (F = 2.61, df = 1, *P* = 0.12), temperature (F = 2.16, df = 1, *P* = 0.16), or their interaction (F = 0.16, df = 1, *P* = 0.7) (Fig. 2d).

**Table 1:** Factorial MANOVA for the treatment effects on life-history traits and ZIKV vector competence measurements.

Source	df	Life-history traits				
		Pillai’s trace	*P*	Development time SCC	Pupa-adult molting SCC	Wing length SCC
JHA	3,14	0.99	< .0001	−1.83	−13.30	−0.38
Temperature	3,14	0.99	< .0001	7.93	6.95	4.53
JHA × temperature	3,14	0.62	0.002	−0.001	12.26	−0.42

	df	Vector competence measurements				
		Pillai’s trace	*P*	Infection SCC	Disseminated infection SCC	Transmission SCC
JHA	3,14	0.88	< .0001	4.92	0.20	−0.67
Temperature	3,14	0.94	< .0001	5.23	−0.23	−0.52
JHA × temperature	3,14	0.68	0.0008	4.61	−1.46	0.10

	df	Pillai’s trace	*P*	Body viral load SCC	Leg viral load SCC	Saliva viral load SCC
JHA	3,14	0.38	0.06	0.49	−0.26	0.54
Temperature	3,14	0.19	0.36	0.73	−0.64	0.88
JHA × temperature	3,14	0.02	0.95	0.86	−0.84	0.71

### Vector competence measurements for Zika virus

**Figure 3:**
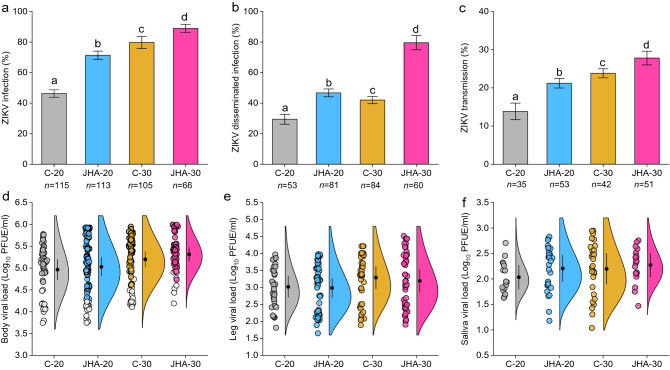
Treatment effects on vector competence measurements for ZIKV, infection (**a**), disseminated infection (**b**), transmission (**c**). Bars represent means ± standard error of the means. Raincloud plots represent ZIKV viral loads in female body (**d**), leg (**e**), saliva (**f**). Each circle in the raincloud plots represents a viral load for an individual ZIKV-infected female. Open circles in (**d**) represent viral loads for females with non-disseminated infection (i.e., ZIKV infection limited to midgut). Black circles inside raincloud represent means ± standard error of the means. Means with different letters indicate statistically significant differences (*P* < 0.05) between each other. Treatments in panels (**d**, **e**, and **f**) were not significantly different (*P* > 0.05) from each other. The number (*n*) of mosquitoes tested is indicated below each bar. Vector competence measurements for ZIKV were determined after 14 days post blood feeding.

Adult mosquito responses to infection of ZIKV following juvenile exposure to JHA under different temperatures were determined for 399 females after 14 days post blood feeding. Factorial MANOVA showed significant effects of JHA, temperature, and their interaction on adult ZIKV infection, disseminated infection, and transmission rates, whereas body, leg, and saliva viral loads were not significantly impacted by treatments (Table 1). Standardized canonical coefficients revealed that ZIKV infection contributed more to the multivariate effects than disseminated infection and transmission (Table 1). Juvenile hormone analog enhanced vector competence at both temperatures, whereas the enhancement of vector competence was higher at 30 °C than 20 °C (Fig. 3a, b, c). The viral loads of ZIKV in body, leg, and saliva were not significantly affected by treatments (Table 1, Fig. 3d, e, f).

## Discussion

Reducing the risk of mosquito-borne pathogens can be achieved by control of the juvenile stages of mosquito vectors using insecticides. Variation in temperature is one of the most ubiquitous environmental factors that can influence juvenile control outcomes by altering mosquito life-history traits and sensitivity to insecticides. Understanding the effects of juvenile exposure to JHA under different temperatures is important given that the application of JHA may occur under a variety of environmental conditions where there is risk of pathogen transmission. However, it is unclear whether temperature interacts with sublethal JHA exposure in modifying mosquito life histories and susceptibility to arbovirus infection and transmission. Identification of how sublethal JHA exposure alters phenotypic traits of mosquitoes may facilitate the development of improved control strategies.

In this study, we measured life-history and vector competence traits in response to sublethal exposure to JHA in cool and warm rearing conditions of the juvenile stages. We demonstrated that JHA, temperature, and their interaction influenced mosquito development rate, pupa-adult molting, size, and vector competence for ZIKV. Specifically, exposure to JHA lengthened development time and enhanced ZIKV infection among mosquitoes that survived to adulthood. Enhanced competence following exposure to insecticides has been previously observed in several mosquito species. For instance, susceptibility to infection with dengue virus (DENV) was increased in *Ae. aegypti* following juvenile exposure to bacterial larvicide, *Bacillus thuringiensis israelensis*^26^. Sindbis virus (SINV) infection and disseminated infection were observed to be high in *Ae. aegypti* that were exposed to malathion during their juvenile stages^7^. Additionally, exposure of adults to bifenthrin enhanced their ZIKV disseminated infection rates in *Ae. albopictus*^44^. Taken together, these observations suggest that insecticide-based control may include acute rapid mortality among targeted mosquitoes; however, individuals that survive the exposure may have enhanced competence for arboviruses which may compromise the efforts to mitigate transmission of arboviruses. Some studies suggested that the naturally occurring insect-specific viruses (ISVs) in wild-caught mosquitoes may modulate their vector competence for arboviruses^45^. However, we did not determine whether our mosquitoes are infected with ISVs and whether the infection would alter the replication or transmission rates of ZIKV in our study. Regardless, the influence of ISVs is not expected to vary by treatment (JHA treatment versus control) and so any bias is expected to have a similar influence on all the mosquitoes.

Development and net growth were altered under different juvenile conditions. Rearing juveniles under low temperature increased development time and sizes of adults, whereas elevated temperature accelerated juvenile development time and produced smaller-sized adults. Exposure to JHA at 20 °C and 30 °C prolonged juvenile development and yielded greater net growth (size) compared to individuals in the controls. Previous studies that determined the effects of temperature and insecticides on mosquitoes observed similar temperature effects; however, exposure to insecticides in those studies accelerated the juvenile development time^7, 46–48^. Variable outcomes of exposure to insecticides on juvenile development time is likely attributable to the mode of action of JHA. Specifically, JHA does not cause mortality among larvae, and so larval density and competition is maintained, leading to increased juvenile development time as a density-dependent effect. The application of traditional insecticides, such as organophosphates, on the other hand, induces larval mortality which can reduce larval density and release surviving larvae from competition for nutrients and space, resulting in acceleration of growth and development^49^.

Increased temperature has been observed to be associated with enhancement of targeted mosquito species sensitivity to insecticides, such as organophosphates and carbamates^7, 50–52^. In our study, pupa-adult molting prevention by JHA in *Ae. aegypti* was observed to be enhanced at elevated temperature. This finding confirms a previous study that detected a positive relationship between prevention of pupa-adult molting by JHA and elevation of temperature in *Cx. pipiens*^30^. Paradoxically, exposure to JHA under high temperature was observed to decrease the toxicity of JHA to juvenile *Ae. aegypti*^31^. This discrepancy in results between our study and others may be due to the use of different mosquito strain and JHA formulation^11^.

Variation of juvenile rearing temperatures produced adults with different sizes at emergence. Variation in adult sizes was associated with changing in mosquito responses to ZIKV infection. Smaller females experienced higher infection and transmission rates for ZIKV compared to larger individuals. Size per se is unlikely to cause enhanced infection, since enhanced arbovirus infection in mosquitoes can be associated with both larger and smaller-sized individuals attributable to different juvenile stressors^7^. However, larger-sized adults may ingest more virus associated with larger blood meals resulting in larger inoculation and dose-dependent infection. It is possible that alteration in mosquito vector competence for ZIKV under different ambient temperatures is associated with variation in sizes of adults and physiological changes that may cause changes in mosquito responses to arboviral infection. For example, elevated rearing conditions, including thermal extremes associated with expression of heat shock proteins, are associated with enhanced *Flavivirus* (DENV)^53^ and *Alphavirus* (SINV) infection in mosquitoes^7, 53^. In contrast, cooler temperatures may result in differential expression of immunoresponsive genes (e.g., RNA interference pathways in *Ae. aegypti* and *Ae. albopictus*)^54, 55^ and be correlated with enhanced vector competence (*Cx. tarsalis* and Western equine encephalitis virus^56^, *Cx. annulirostris* and Murray Valley encephalitis virus^57^, *Ae. albopictus* and chikungunya virus^58^.

Processes that regulate populations, including mosquitoes, act in a density-dependent manner so that birth and death rates change in accordance with whether densities are above or below an equilibrium level^59^. Control practices aim to reduce mosquito population size by inducing mortality at juvenile or adult stages. Here, we show that a 50% reduction in recruitment to adulthood in juvenile treatments exposed to JHA at either temperature did not significantly alter estimates of population growth (λ') compared to controls. Conversely, the surviving mosquitoes benefited from more rapid development and larger-sized adults, which were more competent vectors of ZIKV. These observations underscore the importance of implementation of control practices that minimize sublethal effects attributable to exposure to modest doses of insecticides or insecticide resistance. Whether or not the lifespan of adults, and associated extrinsic incubation period of ZIKV, is altered following sublethal juvenile exposure still needs to be determined. Further empirical and theoretical studies are needed to integrate the impacts of JHA and other insecticides on mosquito population growth, mosquito infectivity, and risk of pathogen transmission.

## Conclusion

We showed that juvenile exposure to JHA not only prevents pupa-adult molting, but also alters important life-history traits of mosquitoes, including ZIKV infection process. Variation in temperature modified the effects JHA on juvenile and adult mosquitoes. Understanding mosquito responses to JHA under a verity of environmental temperature conditions on juvenile and adult life-history traits and vector competence is critical to predict the overall results of vector control. The JHA-enhanced vector competence of adults for ZIKV (or any potential local arbovirus) should be taken into account when use of JHA in vector control. Our findings reveal the needs for considering the interaction between vector control practices and environmental factors on the epidemiology of arboviruses.

## Data Availability

The data that support the findings of this study are included within the article. Additional data are available upon request from the corresponding author.
